# Co-expression of mitosis-regulating genes contributes to malignant progression and prognosis in oligodendrogliomas

**DOI:** 10.18632/oncotarget.5499

**Published:** 2015-10-12

**Authors:** Yanwei Liu, Huimin Hu, Chuanbao Zhang, Haoyuan Wang, Wenlong Zhang, Zheng Wang, Mingyang Li, Wei Zhang, Dabiao Zhou, Tao Jiang

**Affiliations:** ^1^ Department of Molecular Neuropathology, Beijing Neurosurgical Institute, Capital Medical University, Beijing, China; ^2^ Department of Neurosurgery, Beijing Tiantan Hospital, Capital Medical University, Beijing, China; ^3^ Department of Neurosurgery, Zhujiang Hospital, Southern Medical University, Guangzhou, China; ^4^ Brain Tumor Center, Beijing Institute for Brain Disorders, Beijing, China; ^5^ Chinese Glioma Cooperative Group (CGCG), China; ^6^ National Clinical Research Center for Neurological Diseases, Beijing, China

**Keywords:** oligodendrogliomas, prognosis, malignant progression, proliferation, gene expression

## Abstract

The clinical prognosis of patients with glioma is determined by tumor grades, but tumors of different subtypes with equal malignancy grade usually have different prognosis that is largely determined by genetic abnormalities. Oligodendrogliomas (ODs) are the second most common type of gliomas. In this study, integrative analyses found that distribution of TCGA transcriptomic subtypes was associated with grade progression in ODs. To identify critical gene(s) associated with tumor grades and TCGA subtypes, we analyzed 34 normal brain tissue (NBT), 146 WHO grade II and 130 grade III ODs by microarray and RNA sequencing, and identified a co-expression network of six genes (*AURKA*, *NDC80*,*CENPK*, *KIAA0101*, *TIMELESS* and *MELK*) that was associated with tumor grades and TCGA subtypes as well as Ki-67 expression. Validation of the six genes was performed by qPCR in additional 28 ODs. Importantly, these genes also were validated in four high-grade recurrent gliomas and the initial lower-grade gliomas resected from the same patients. Finally, the RNA data on two genes with the highest discrimination potential (*AURKA* and *NDC80*) and Ki-67 were validated on an independent cohort (5 NBTs and 86 ODs) by immunohistochemistry. Knockdown of *AURKA* and *NDC80* by siRNAs suppressed Ki-67 expression and proliferation of gliomas cells. Survival analysis showed that high expression of the six genes corporately indicated a poor survival outcome. Correlation and protein interaction analysis provided further evidence for this co-expression network. These data suggest that the co-expression of the six mitosis-regulating genes was associated with malignant progression and prognosis in ODs.

## INTRODUCTION

Oligodendrogliomas (ODs) are the second most common type of gliomas and classified as oligodendroglioma gradeII (OII), anaplastic oligodendroglioma grade III (OIII), oligoastrocytoma grade II (OAII) or anaplastic oligoastrocytoma grade III (OAIII), according to the World Health Organization (WHO) classification system [1]. This grading is based on the presence or absence of nuclear atypia, mitoses, vascular proliferation and necrosis. Increasing grades of ODs are reflected by an elevated level of abnormal cytology resulting from genetic abnormalities. At present, loss of heterozygosity of the chromosomal arms 1p and 19q (LOH1p/19q) and *IDH* (isocitrate dehydrogenase) mutation are the most important candidate markers to guide treatment decisions in ODs.

LOH1p/19q occurs in 50%–70% of ODs, especially in the low-grade type tumors [2]. This finding may have implications for putative tumor suppressor gene(s) located in this region and provide important insights into the pathogenesis of ODs. Indeed, many tumor suppressors located on chromosomal arms 1p/19q were identified by examining the comprehensive gene expression profiles [3, 4]. These silencing genes by LOH1p/19q may play tumorigenic roles in ODs. Recently, *isocitrate dehydrogenase 1/2 (IDH1/2)* was shown to be mutated in up to 70%–80% of low-grade gliomas (grades II and III) [5]. IDH1 catalyzes the oxidative decarboxylation of isocitrate to α-ketoglutarate (α-KG). This mutation occurs at the arginine residue of the enzyme's active site and causes a gain of function, leading to a reduction of α-KG to D-2-hydroxyglutarate (D-2HG) [6]. Accumulation of this oncometabolite induces DNA hypermethylation, leading to genome-wide epigenetic changes and predisposing cells toward neoplastic transformation [7]. Mutations in *IDH1* have shown a prognostic value. High frequency of the *IDH1* mutation and LOH1p/19q in ODs suggests critical roles in early tumor development [5, 8]. In addition, *ATRX* mutation and *TERT* promoter mutation were identified as biomarker in lower-grade gliomas [9, 10]. Relative to astrocytomas, *ATRX* mutation rarely occurs in ODs [9, 10]. *TERT* promoter mutations occur in 79.3% of oligodendrogliomas, but occur less frequently in astrocytomas or oligoastrocytomas. Interestingly, the *TERT* promoter mutation always occurred in the setting of patients with *IDH1/2* mutation [9, 10] and this mutation highly correlated with upregulated *TERT* mRNA expression and tumor grade in adult gliomas [11, 12]. However, all of these gene alterations have not been proven to be the driver events in promoting malignant progression of ODs.

A robust gene expression-based molecular classification is able to group GBM (glioblastoma multiforme) into proneural, neural, classical and mesenchymal subtypes [13]. Recently, there have been several reports that the TCGA molecular subtypes of glioblastoma are relevant to lower grade gliomas [14–16]. In this study, we evaluated the relevance of the TCGA subtypes to ODs and found that the distribution of TCGA subtypes was associated with ODs grades. To identify genes driving malignant progression among the different grades and subtypes, we analyzed the data from whole-genome expression by microarray and RNA-seq in four datasets (CGGA, TCGA, GSE16011 and REMBRANDT). We identified a co-expression network of six new genes that was associated with malignant progression in different grades and TCGA subtypes of ODs. This co-expression network was shown to play an important role in the regulation of cell proliferation and was associated with a poor survival outcome in patients with ODs. These genes represent new opportunities for understanding the fundamental basis for malignant progression of ODs as well as novel interfering targets of ODs.

## RESULTS

### TCGA subtypes were associated with grade progression of ODs

*IDH1/2* mutation and LOH 1p/19q are the most important candidate markers to guide treatment decisions in ODs. We collected 373 ODs with the information on *IDH1/2* and 1p/19q from CGGA dataset (Supplementary Figure 1A) and identified mutations in *IDH1* in more than 70.5% (263/373) and *IDH2* in 4.1% (14/345) of WHO grade II and III ODs. LOH1p/19q was identified in 39.4% (147/373) of ODs. We found that the high frequency of these markers is already present in ODII (75% in *IDH1*, 5.1% in *IDH2* and 46.0% in LOH1p/19q) while the frequency does not increase in ODIII (61.6% in *IDH1*, 1.8% in *IDH2* and 26.4% in LOH1p/19q). The result suggests that they may not be associated with malignant progression from ODII–ODIII.

**Figure 1:**
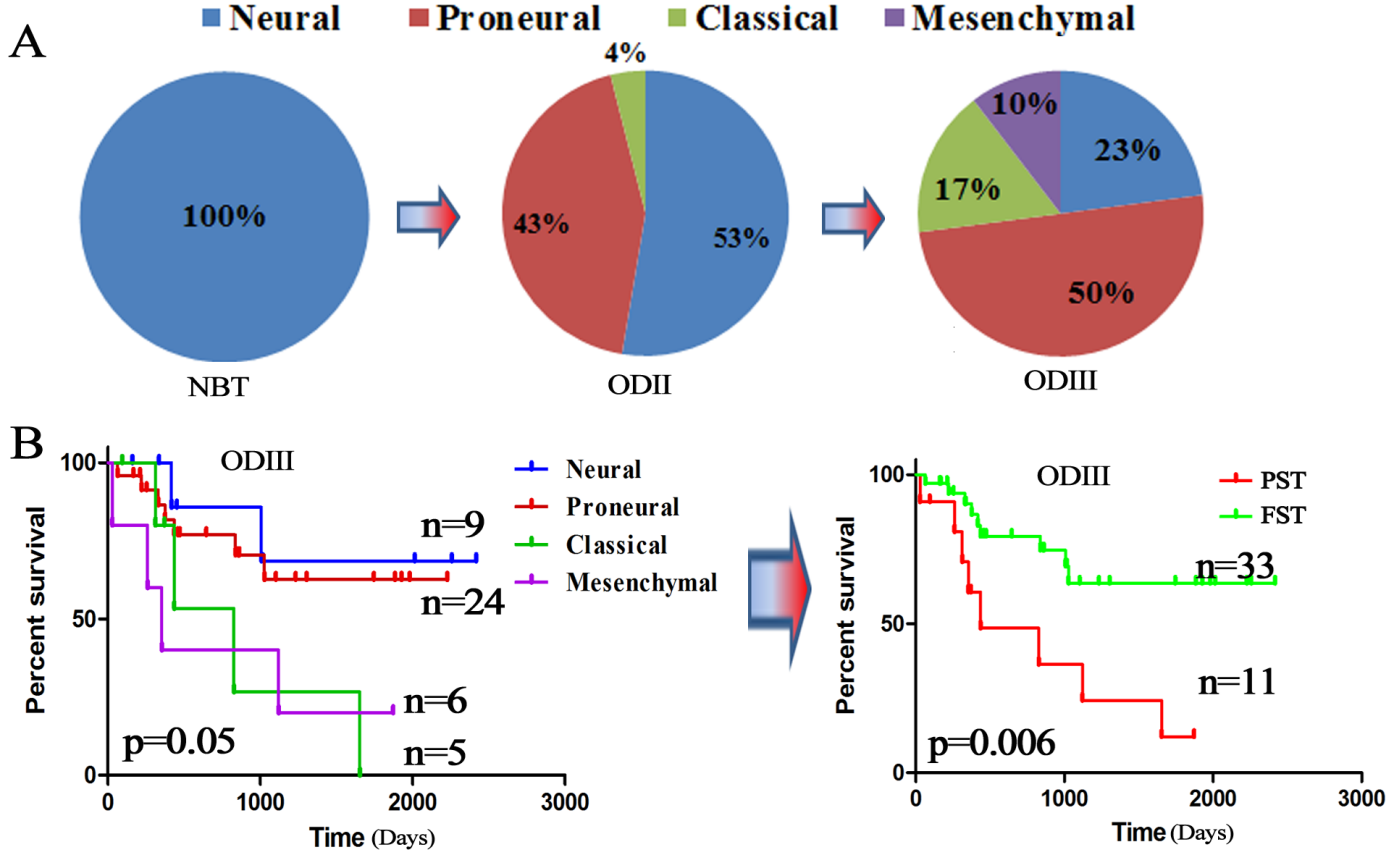
TCGA subtypes were associated with OD grades **A.** The distribution of the TCGA subtypes in different grades of ODs from the CGGA dataset (5 NBTs, 76 ODIIs and 48 ODIIIs); **B.** Kaplan–Meier survival analysis divided the TCGA subtypes into a favorable survival subtype (FST: neural and proneural) and a poor survival subtype (PST: classical and mesenchymal) based on the CGGA dataset.

A robust gene expression-based molecular classification is able to group GBM into proneural, neural, classical and mesenchymal subtypes [13, 14–16]. In this study, we found that the distribution of the TCGA subtypes varied significantly in different grades of ODs. As showed in Figure 1A, the TCGA subtypes gradually transformed from neural and proneural subtypes to classical and mesenchymal subtypes with increasing tumor grades. The classical subtype, known as the proliferative subtype [17], was not found in normal brain tissue (NBT), but then started appearing in ODII (4%) and ODIII (17%). The same distributions were also found in both the GSE16011 and REMBRANDT datasets (Supplementary Figure 1B). Patients with the neural and proneural subtypes displayed significantly improved survival compared those with the classical and mesenchymal subtypes [15, 18–19] (Figure 1B). Therefore, we classified the neural and proneural subtypes as favorable survival subtype (FST) and the classical and mesenchymal subtypes as poor survival subtype (PST). These data showed that the TCGA subtypes gradually transformed from FST to PST as the tumor grades increased.

### Identifying candidate genes promoting grade progression

**Figure 2:**
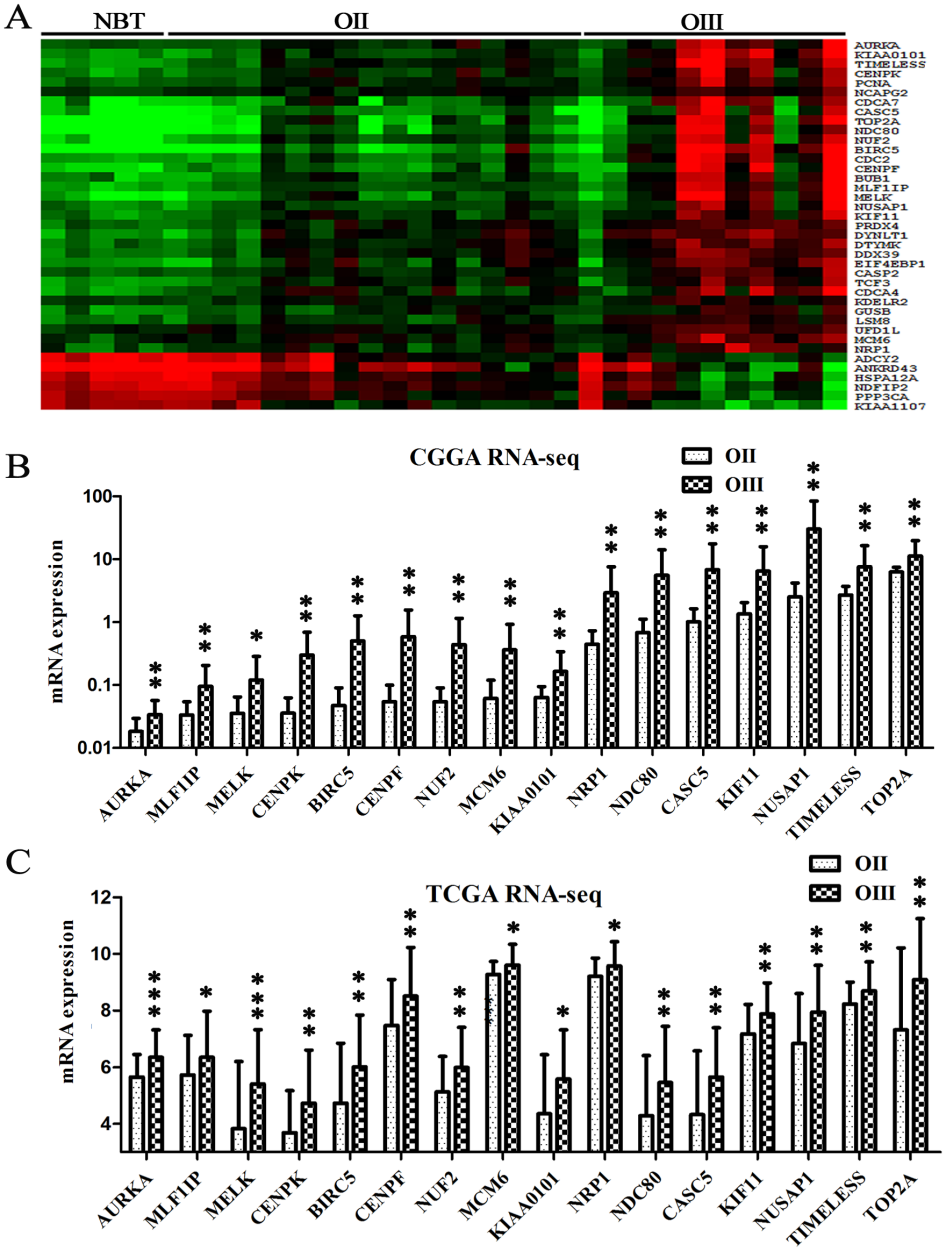
Identifying candidate genes associated with grade progression **A.** Transcripts levels of 39 candidate genes were identified by microarray from the CGGA dataset (5 NBTs, 17 OIIs and 11OIIIs) which showed either gradual increases (33 genes) or decreases (6 genes) in the entire grade progression from NBT to OII and OIII (*p* < 0.05); The 39 candidate genes were obtained by overlapping data analysis from CGGA, GES16011 and REMBRANDT datasets. **B.** 16 candidate genes were further confirmed by the RNA-seq data of the CGGA dataset (27 OIIs and 11 OIIIs); **C.** The 16 candidate genes were validated on an independent RNA-seq data of the TCGA dataset (64 OIIs and 41 OIIIs).

To identify critical genes that were associated with grade progression, microarray data for 167 samples (34 NBTs, 55 OIIs, and 78 OIIIs) were used to compare the genome expression in three datasets (CGGA, GSE16011 and REMBRANDT). To avoid discrepancies due to the different platforms, the data were analyzed separately within each dataset (Supplementary Table 1). Overlapping the results from the three datasets, a total of 2842 upregulated and 2624 downregulated genes were identified in OII compared with NBT. 401 upregulated and 54 downregulated genes were identified in the transition from OII to OIII. (Supplementary Figure 2A and Supplementary Table 2). However, 33 upregulated and 6 downregulated candidate genes were identified over the entire gradual process of malignant progression from NBT to OII–OIII (Figure 2A). To avoid any confusion in the data induced by a gene assessed with more than one probe, the probes used were consistent from NBT to OII to OIII. Finally, 16 out of 33 upregulated genes (none of 6 downregulated) were further confirmed by RNA-seq in 38 samples of the CGGA dataset (27 OIIs and 11 OIIIs) (Figure 2B). Furthermore, the 16 genes were validated in another independent cohort consisting of 105 samples from the TCGA dataset (64 OIIs and 41 OIIIs) (Figure 2C). A gene-set enrichment analysis of these upregulated genes showed that cancer associated pathways and the cell cycle were the main upregulated pathways in the grade progression of ODs (Supplementary Figure 2B). Kaplan–Meier survival analysis showed that almost all of these candidate genes (except for *NRP1*, *p* = 0.43) were significantly associated with poor survival outcomes in patients with OIII (*p* < 0.05) (Supplementary Figure 3).

### Identifying candidate genes associated with distribution of TCGA subtypes

**Figure 3:**
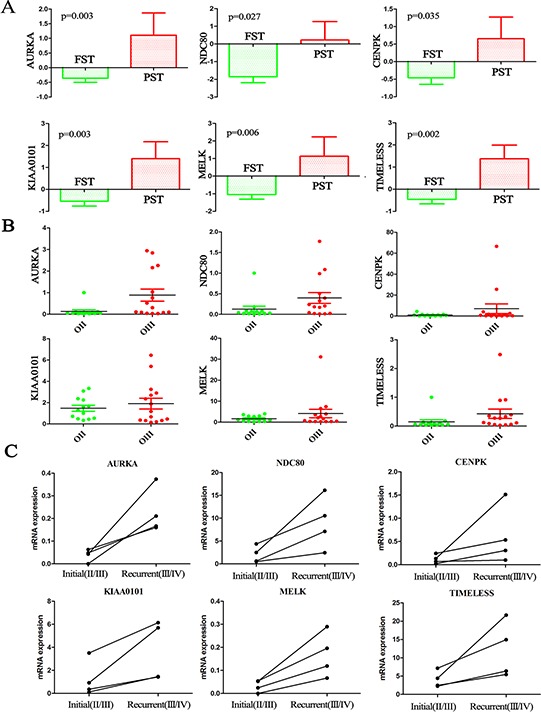
Candidate genes associated with TCGA subtypes were validated on additional samples **A.** The expression levels of the six candidate genes showed significant differences between the FST and PST in the CGGA dataset (*p* < 0.05); **B.** The expression levels of the six genes were higher in OIII than in OII as assessed by qPCR on the additional CGGA samples (13 OIIs and 15 OIIIs). **C.** The expression levels of the six genes were significantly increased in four high-grade recurrent gliomas (AOA/sGBM, grade III/IV) compared with the initial lower-grade gliomas (grade II/III) resected from the same patients. The four paired samples: OA progress to AOA; OA progress to sGBM; AO progress to sGBM; AA progress to sGBM.

Given the finding that the distribution of TCGA subtypes significantly changed with the increasing tumor grades, we found that 6 out of the 16 candidate genes displayed differential expression levels between the FST and PST in the CGGA dataset (Figure 3A). The result was further validated in the GSE16011 and REMBRANDT datasets (Supplementary Figure 4A and 4B). The six genes were *AURKA* (*aurora kinase A*)*, NDC80* (*highly expressed in cancer 1*)*, KIAA0101, TIMELESS* (*timeless circadian clock*)*, MELK* (*maternal embryonic leucine zipper kinase*)*,* and *CENPK* (*centromere protein K*). Previous studies have suggested that the six genes, such as *AURKA*, play important roles in the regulation of cell mitosis, cell cycle and proliferation [20–24], which is consistent with the above data analysis that cell proliferation and cell cycle were the main upregulated pathways in the grade progression of ODs.

### Candidate genes were further validated in 28 samples and 4 paired samples

Validation of the six genes was performed on 13 OIIs and 15 OIIIs from the CGGA by RT-PCR. The results corresponded well to the microarray and RNA-seq data that showed expression levels of the six genes were higher in OIII than in OII (Figure 3B). Secondary GBM (sGBM) develop slowly through progression from grade II and III diffuse gliomas. We analyzed four paired samples with initial gliomas (grade II/III) and recurrent gliomas (grade III/sGBM) by RNA sequencing. As expected, all of the six genes were significantly increased with increasing tumor grades from the initial lower-grade gliomas to the recurrent high-grade gliomas in the same patients (Figure 3C). Moreover, ki-67 protein of the recurrent tumors is higher than those observed in the initial tumors (*p* = 0.02) (Supplementary Figure 4C).

### Co-expression of the six candidate genes was associated with cell proliferation

**Figure 4:**
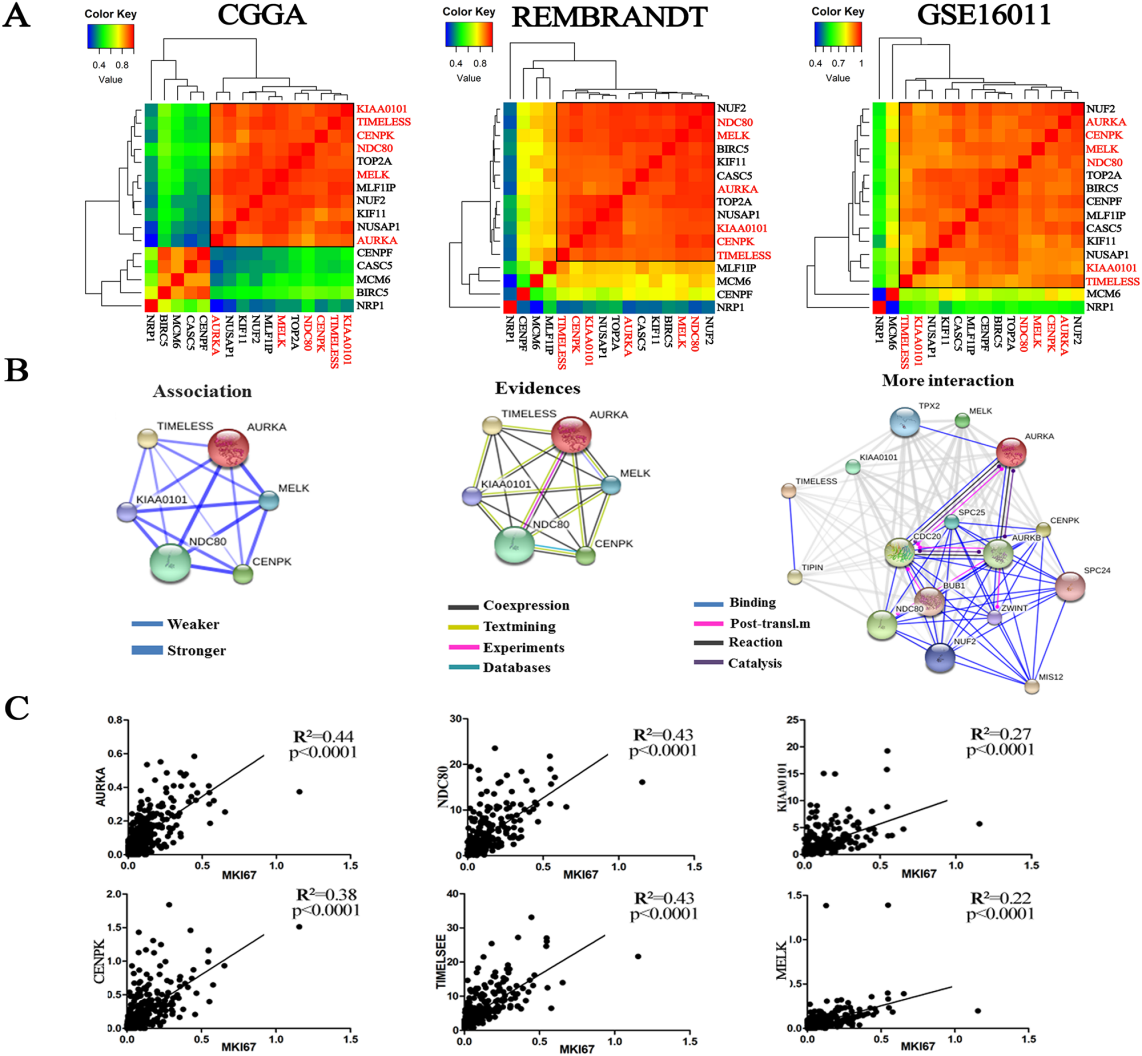
Co-expression of the six genes was associated with Ki-67 **A.** Correlation analysis using the 16 candidate genes in the three datasets revealed a subclass that is characterized by the six genes (CGGA: 5 NBTs, 17 OIIs and 11 OIIIs; REMBRANDT: 21 NBTs, 30 OIIs, 23 OIIIs; GSE16011: 8 NBTs, 8 OIIs, 44 OIIIs); **B.** The protein-protein interactions of the six candidate genes were predicted from an online database (STRING); **C.** The expression levels of the six genes showed a significant positive correlation with Ki-67 expression from the RNA-seq data of the CGGA dataset (109 grade II, 72 grade III and 144 grade IV gliomas).

To further investigate this interworking network composed of the six genes, a correlation analysis of the 16 candidate genes was conducted in three datasets, which revealed a subclass that was characterized by the six genes (Figure 4A). Three datasets consistently showed that there was a strong correlation of the six genes (coefficient value >0.8). In addition, we predicted the protein-protein interactions from an online database (STRING: http://string.embl.de/), which surprisingly showed direct (physical) and indirect (functional) interactions for all of these six genes (Figure 4B). On loosening the limitations to allow for more interactions, *BUB1* (*mitotic checkpoint serine/threonine kinase*) and *NUF2* (*NDC80 kinetochore complex component*) became part of this regulatory network. In the above analysis, *BUB1* and *NUF2* were also found to be associated with tumor grades. Given that the association between the six genes and cell proliferation, the RNA-seq data in the 325 glioma samples of the CGGA dataset was analyzed and showed that the expression levels of the six genes were positively correlated with the proliferative marker (Ki-67) (Figure 4C).

### Validation of the RNA data by immunohistochemistry (IHC)

**Figure 5:**
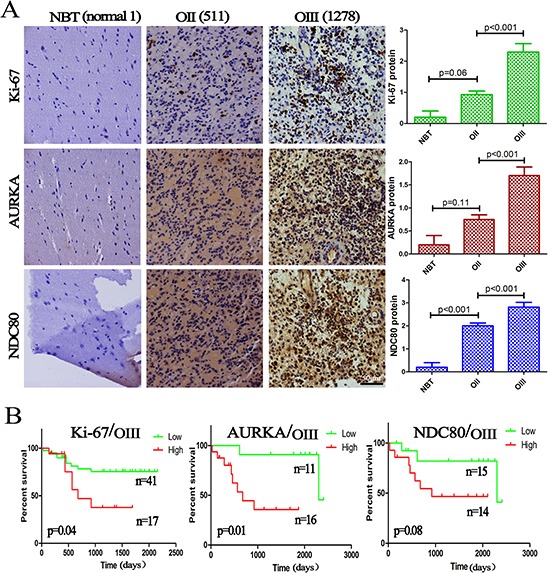
Validation of the RNA data by IHC **A.** The protein expression of Ki-67, AURKA and NDC80 was significantly increased with increasing OD grades, and semiquantitative analysis was performed in 5 NBTs, 57 OIIs and 29 OIIIs; **B.** Kaplan–Meier survival analysis showed that high expression of these three proteins indicated a poor survival. The columns represent the same samples; 511 and 1278 refer to the sample ID in the CGGA dataset.

The differential expression of 2 genes (*AURKA* and *NDC80*) with the highest discrimination potential in the co-expression network were further validated using IHC in a series of paraffin sections from the CGGA (5 NBTs, 57 OIIs and 29 OIIIs). Simultaneously, IHC on Ki-67 was also performed to observe cell proliferation in the same samples. As expected, an increasing level of AURKA and NDC80 as well as Ki-67 protein was associated with increasing tumor grades (Figure 5A). Importantly, as showed in Figure 5B, we were once again reminded that the high expression of these three proteins indicated a poor survival outcome (Ki-67 at *p* = 0.04; AURKA at *p* = 0.01; and NDC80 at *p* = 0.08).

### Deletion of the candidate genes suppressed Ki-67 and cell proliferation

**Figure 6:**
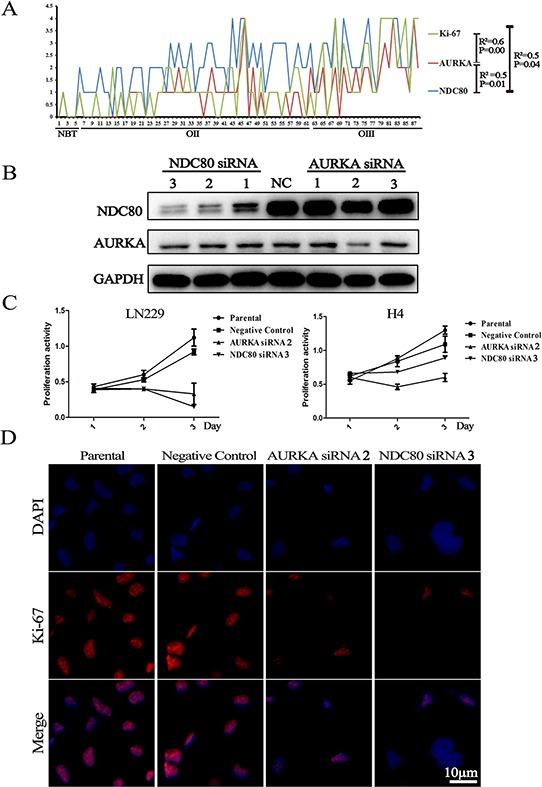
Deletion of the candidate genes suppressed Ki-67 expression and cell proliferation **A.** The three proteins (Ki-67, AURKA and NDC80) showed similar expression levels and significantly increased with increasing tumor grades; **B.** Western blotting showed that suppression of the expression of AURKA and NDC80 with siRNAs significantly decreased their protein expression; **C.** and **D.** MTT assay and cell immunofluorescence showed that suppression of AURKA and NDC80 expression suppressed the malignant proliferation of glioma cells and Ki-67 expression. Parental cells are non-transfected H4 and LN229 cells; Negative Control refers to transfected H4 and LN229 cells with a scrambled siRNA sequence that will not lead to specific degradation of any known cellular mRNA.

In the IHC assay, 88 samples (5 NBTs, 56 OIIs and 27 OIIIs) were available for the simultaneous analysis of the protein expression of Ki-67, AURKA and NDC80. As showed in Figure 6A, the expression of Ki-67 was significantly correlated with AURKA and NDC80 (R^2^ = 0.6, *p* = 0.000 and R^2^ = 0.5, *p* = 0.04, respectively), which was consistent with the RNA data. To confirm the roles of the candidate genes as drivers of proliferation, siRNAs for *AURKA* and *NDC80* were transfected into H4 and LN229 glioma cells with liposome transfection reagents for transient expression. Western blot analysis confirmed the specific knockdown of AURKA and NDC80 (siRNA1, 2, 3) (Figure 6B). As expected, treatment with siRNAs (AURKA siRNA2 and NDC80 siRNA3) significantly decreased the Ki-67 expression and the malignant proliferation of glioma cells (Figure 6C and D). However, knockdown of AURKA (or NDC80) did not affect the NDC80 (or AURKA) expression in glioma cells. In conclusion, the results indicated that these candidate genes were the primary drivers in promoting the malignant proliferation of glioma cells.

## DISCUSSION

The stepwise progression from an early dysplastic lesion to a malignant neoplasm is associated with abnormal gene expression [25]. It is now known that the deregulation of proliferation is caused by the loss of tumor suppressors or the gain of oncogenes. The increasing mitotic activity indicates an increasing malignant degree and malignant tumor subtype transition [26]. ODs are the second most common type of gliomas. In contrast to astrocytic tumors, ODs have a better prognosis associated with LOH1p/19q and *IDH1* mutation. However, these genetic alterations have been widely described as having roles in promoting the development of ODs [27, 28]. However, in our study, they may not be associated with malignant progression from ODII to ODIII and sGBM, as the high frequency of the two gene alterations are already present in ODII and the frequency does not significantly increase and even decreases in ODIII. In the present study, we identified, by RNA-seq and microarray, a co-expression network of six mitosis-associated genes that may promote grade progression and the malignant transition of TCGA subtypes in ODs. All six of the candidate genes were closely related to cell mitosis and high cellular aneuploidy. Importantly, these six genes showed an increased expression along with increasing tumor grades in the same patients. Suppression of the expression of the candidate genes suppressed Ki-67 and glioma cell proliferation. We concluded that interactions among the six genes complement and assist each other to promote abnormal mitoses and malignant proliferation, resulting in the malignant progression of the ODs.

AURKA, a cell cycle-regulated kinase, plays multiple roles in the regulation of cell division. The most important roles include entry into mitosis, assembly of the microtubule spindle and completion of cytokinesis [29]. AURKA, combined with another crucial mitotic kinase, PLK1, activates the CDK-activating phosphatase CDC25B, leading cells into mitosis [30]. Overexpression of AURKA induces aneuploidy and chromosomal instability and overrides the mitotic spindle checkpoint, driving progression in head and neck cancer [31]. This results in tetraploid cells with an increased centrosome number that continue to divide without a functioning G1 checkpoint and TP53 control, which in turn play a leading role in the pathogenesis of various tumors. An increased level of AURKA mRNA was associated with an overall decrease in the survival rate. Thus, AURKA has been used as a potential target for the development of tumor-targeted therapeutics [32]. NDC80, also called as Hec1 (highly expressed in cancer 1), is involved in the organization and stabilization of microtubule-kinetochore interactions and is required for proper chromosome segregation [33]. Overexpression of NDC80 was found to be responsible for hyper-activating the mitotic checkpoint and inducing tumor formation [21]. Interestingly, Bièche and his team (2011) reported that the mRNA expressions of NDC80 and AURKA were simultaneously higher in breast tumors than in the normal tissues [34]. In the present study, a prediction of protein-protein interactions showed a close association between AURKA and NDC80.

TIMELESS, a circadian clock protein, is highly conserved and also forms a core component of the cell cycle checkpoint system [35]. A recent study showed that a higher level of TIMELESS in colorectal cancer tissue is associated with TNM stages III-IV and microsatellite instability, which relates to the survival of patients [36]. KIAA0101, a proliferating cell nuclear antigen (PCNA)-associated factor, is involved in the regulation of DNA repair, cell cycle progression and cell proliferation. Overexpression of KIAA0101 promotes the progression of a variety of tumors and has a poor prognosis [23]. Importantly, PCNA is also on the list of upregulated candidate genes in our study. The other two driver genes, CENPK and MELK, are also critical for the regulation of cell proliferation. CENPK, a centromere protein, is required for proper kinetochore function and mitotic progression and appears to work in coordination with the NDC80 kinetochore complex [20]. MELK, a candidate oncogene, is highly expressed in glioblastomas and contributes to tumor growth [37]. Other molecules, such as BUB1 and NUF2, were found to be involved in the mitosis-regulated network. BUB1 possibly functions in the DNA damage response, and mutations in this gene have been associated with aneuploidy and several forms of cancer [38]. Interestingly, NUF2 is a component of the NDC80 kinetochore complex and has been found to be associated with the centromeres of mitotic HeLa cells [39].

These candidate genes experienced direct and indirect interactions that regulate cell mitosis and cell cycle. In short, all of these genes form an effective mitotic control network that contributes to the malignant progression of ODs. The co-expression network of six mitosis-related genes may reflect critical processes in the malignant progression of ODs (grade progression and subtype transitions). However, the interactions among these genes undergoing dysregulated expression are likely to be more complex than previously thought, and greater insights into their mechanisms are required for a better understanding of their function.

## MATERIALS AND METHODS

### Patients and samples

Tumor specimens were obtained from patients who underwent positive debulking surgery in the Neurosurgery Department of Beijing Tiantan Hospital from 2006 to 2014. These samples were used to perform mRNA expression profiling, RNA sequencing, immunohistochemistry and survival analysis. The number of samples in each analysis is outlined in the following sections. Normal adult brain samples were obtained after informed consent from patients with severe traumatic brain injury who required post-trauma surgery or from patients who had undergone surgery for primary epilepsy. Patients were eligible for the study if their diagnosis was established histologically by 2 neuropathologists according to the 2007 WHO classification guidelines. This study was approved by the institutional review boards of all of the hospitals involved in the study, and wrote informed consent was obtained from all patients.

### RNA extraction

All tissue samples were immediately snap-frozen in liquid nitrogen after surgery. A hematoxylin and eosin–stained frozen section was prepared from each sample to assess the percentage of tumor cells before RNA extraction. Only samples with 80% tumor cells were selected for RNA extraction. Total RNA was extracted using the mirVana miRNA Isolation kit (Ambion) according to the manufacturer's protocol. RNA concentration and quality were measured using the NanoDrop-2000C spectrophotometer (NanoDrop Technologies)

### Microarray data

Microarray analysis was performed using the Agilent Whole Human Genome Array according to the manufacturer's instructions [18]. The integrity of total RNA was checked using an Agilent 2100 Bioanalyzer (Agilent). cDNA and biotiny-lated cRNA were synthesized and hybridized to the array. Data were acquired using the Agilent G2565BA Microarray Scanner System and Agilent Feature Extraction Software (version 9.1). Probe intensities were normalized using GeneSpring GX 11.0. The microarray data was deposited in the CGGA (http://www.cgga.org.cn). The REMBRANDT and GSE16011 microarray data were downloaded from the repository for molecular brain neoplasia data (https://caintegrator.nci.nih.gov/rembrandt/) and the Gene Expression Omnibus (http://www.ncbi.nlm.nih.gov/geo/).

### RNA-sequencing data

RNA-sequencing was performed on 325 glioma samples as previously reported [40]. Briefly, only high quality sample with RNA Integrity Number (RIN) value greater than or equal to 7.0 was used to construct sequencing library. The sequencing libraries were sequenced on the Illumina HiSeq 2000 platform using the 101-bp pair-end sequencing strategy. Short sequencereads were aligned to the human reference genome (Hg19Refseq) using the Burrows-Wheeler Aligner (BWA, Version 0.6.2-r126). The TCGA RNA sequencing data were downloaded from the TCGA database (http://cancergenome.nih.gov).

### qPCR

The expression levels of the candidate genes in the samples were analyzed with real-time quantitative PCR using the SYBR Supermix Kit (Bio-Rad, Hercules, CA). PCR included the following components: 100 nM of each primer, diluted cDNA templates and the iQ SYBR Green supermix. PCR efficiency was examined by serially diluting the template cDNA, and the melting curve data were collected to check PCR specificity. Each cDNA sample was run in triplicate. The ACTIN primer was included on every plate to avoid sample variations. The relative mRNA level was presented as unit values of 2^ [Ct (ACTIN)–Ct (gene of interest)]. The primers used are listed in Supplementary Table 3.

### Immunohistochemistry (IHC)

Immunohistochemistry was performed on 3 tissue microarrays (5 NBTs, 57 OIIs and 29 OIIIs). Briefly, surgical specimens were fixed in formalin, routinely processed and embedded in paraffin. Five micron-thick sections were prepared, and immunohistochemical staining with a streptavidin-biotin immunoperoxidase assay was performed using polyclonal antibodies to Ki-67 (Cell Signaling Technology, USA), AURKA and NDC80 (Protein Tech Group, China). The staining intensity was jointly scored by two pathologists without any knowledge of the clinical information. The proportion of positively stained tumor cells was graded as follows: 0, no or very few positive tumor cells; 1, moderate positive tumor cells; and 2, mass positive tumor cells. The intensity of staining was recorded on a scale of 0 (no or very weak staining staining), 1 (moderate staining) and 2 (strong staining). The staining index was calculated as follows: staining index = staining intensity × proportion of positively stained tumor cells. The staining intensity, proportion were adjusted according for each antibody.

### Cell culture and proliferation assay

Human LN229 and H4 gliomas cells were obtained from the China Academia Sinica Cell Repository, Shanghai, China. The cells were maintained in Dulbecco's modified Eagle's medium (Gibco, Los Angeles, CA, USA) supplemented with 10% fetal bovine serum (Gibco) and were incubated at 37°C in a 5% CO_2_ atmosphere. Following transfection with *AURKA* and *NDC80* siRNAs, an MTT (3-(4,5-Dimethylthiazol-2-yl)-2,5-diphenyltetrazoliumbromide) assay was used to quantitate the cell viability. The sequences of the siRNAs (GenePharma, Shanghai, China) are : *AURKA* siRNA2 sense: 5′-GCAUUUCAGGACCUGUUAATT-3′, antisense: 5′-UUAACAGGUCCUGAAAUGCTT-3′; *NDC80* siRNA3 sense: 5′-GC AGCCUUAGUUUGGCUAATT-3′, antisense: 5′-UUAGCCAAACUAAGGCUGCT T-3′; Negative Control sense: 5′-UUCUUCGAACGUGUCACGUTT-3′, antisense:

5′-ACGUGACACGUUCGGAGAATT-3′. Cells (2000 cells/well) were plated in 96-well plates and incubated at 37°C overnight. The siRNAs (50 nmol/L) (Genepharma, China) were transfected into cells using Lipofectamine 2000 (Invitrogen, USA) according to the manufacturer's protocol. For the controls, either cells transfected with a negative control or untransfected parental cells were used. The absorbance values of each well were measured with a microplate spectrophotometer (TECAN, Infinite M200PRO) at 490 nm.

### Western blot

Whole-cell lysates were prepared using RIPA buffer (Cell Signal Technology). Equal amounts of total protein (30 μg) from the cell lysates were loaded onto a 10% SDS/PAGE gel, transferred to a PVDF membrane (Millipore) and detected using an ECL western blotting Detection System (Biorad). Primary rabbit polyclonal antibodies against AURKA (Proteintech Group, Inc., Catalog No.: 10297–1-AP, 1:200) and NDC80 (Proteintech Group, Inc., Catalog No.: 18932–1-AP, 1:500) were used. An antibody against GAPDH (Proteintech Group, Inc., Catalog No.: 60004–1-Ig, 1:5000) was used as the loading control. Goat anti-rabbit IgG-HRP and goat anti-mouse IgG-HRP (Zhongshan Gold Bridge Biotechnology, 1:5000) were used as secondary antibodies.

### Immunofluorescence for cultured cells

Cells were grown on glass coverslips (BD Biosciences, Bedford, MA, USA) overnight to 70% confluence. Coverslips with cells were fixed with a formalin solution for 10 min. Coverslips were then rinsed twice in phosphate-buffered saline (PBS), pH 7.4, and incubated for 10 min in blocking solution (1% bovine serum albumin in PBS) before antibody incubation. A primary antibody against Ki-67 (1:200; CST) was used. The slides were then incubated overnight at 4°C. Dylight 594 AffiniPure Goat Anti-Rabbit lgG (H+L) (EarthOx, USA) was used as secondary antibody at a 1:400. Cells were counterstained with mounting media containing DAPI.

### Statistical analysis

We applied the classification system of the TCGA to our CGGA samples. Genes were ordered using the predictive 840-gene list of the TCGA classification system, and the annotations of CGGA samples were derived from the Prediction Analysis of Microarrays classifier [13, 18, 41]. Differentially expressed genes were detected by unpaired Student's *t*-test using SPSS version 13.0. Kaplan-Meier survival analysis was used to estimate the survival distributions. The log-rank test was applied to assess the statistical significance between the stratified survival groups using the GraphPad Prism version 4.0 statistical software. The correlations of gene expression were analyzed using *R* × 64 3.0.1 software (Pearson correlation). KEGG pathway maps analysis was performed using DAVID (http://david.abcc.ncifcrf.gov/). Heat maps of different grades of ODs were constructed by Gene Cluster 3.0 and Gene Tree View software. Cut points for the different variables were set according to at the median. A two-sided *p* value <0.05 was regarded as significant.

## SUPPLEMENTARY FIGURES AND TABLES




