# Supplementary material for: Co-expression of mitosis-regulating genes contributes to malignant progression and prognosis in oligodendrogliomas
Source: Oncotarget. 2015 Oct 12;6(35):38257–69. doi: 10.18632/oncotarget.5499 (PMC4741997; doi:10.18632/oncotarget.5499)
Supplement: Supplementary file 1 [file oncotarget-06-38257-s001.pdf]

SUPPLEMENTARY FIGURES AND TABLES

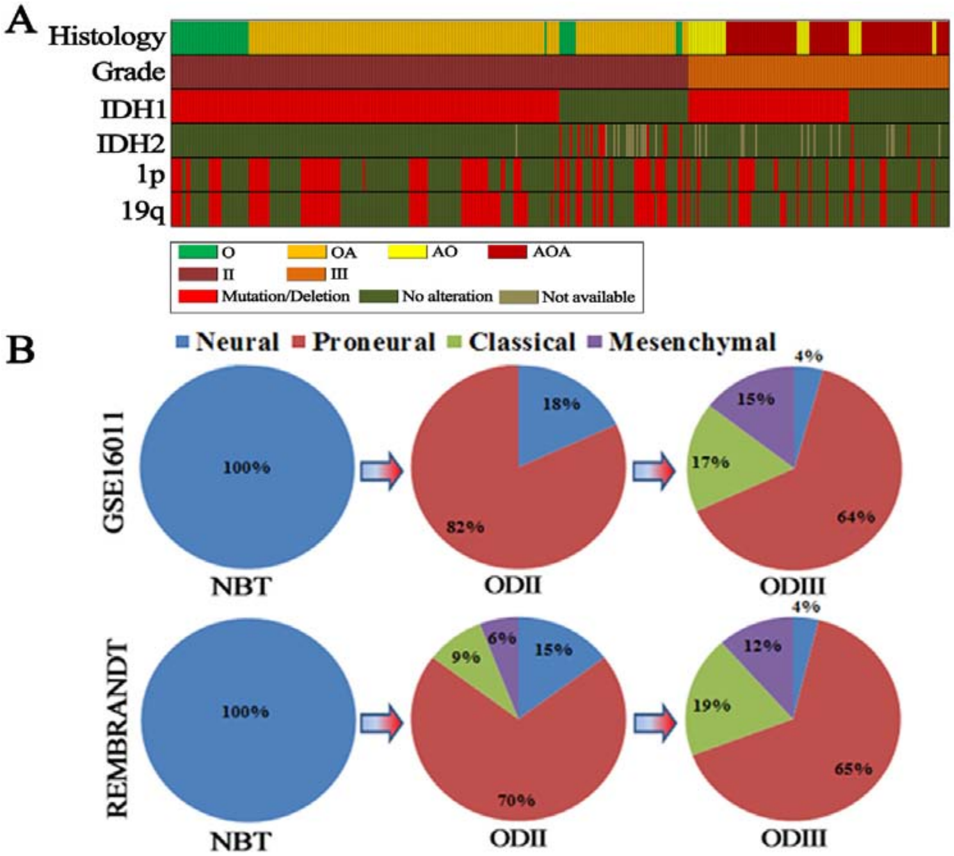

Supplementary Figure S1: Overview of molecular aberrations and molecular classification in ODs. A. Distribution of *IDH1/2* and LOH1p/19q in ODs of CGGA dataset (248 ODIIs and 125 ODIIIs). **B.** Distribution of the TCGA subtypes in different grades of ODs from the GSE16011(8 NBTs, 8 ODIIs and 44 ODIIIs) and REMBRANDT (21 NBTs, 30 ODIIs and 23 ODIIIs) datasets.

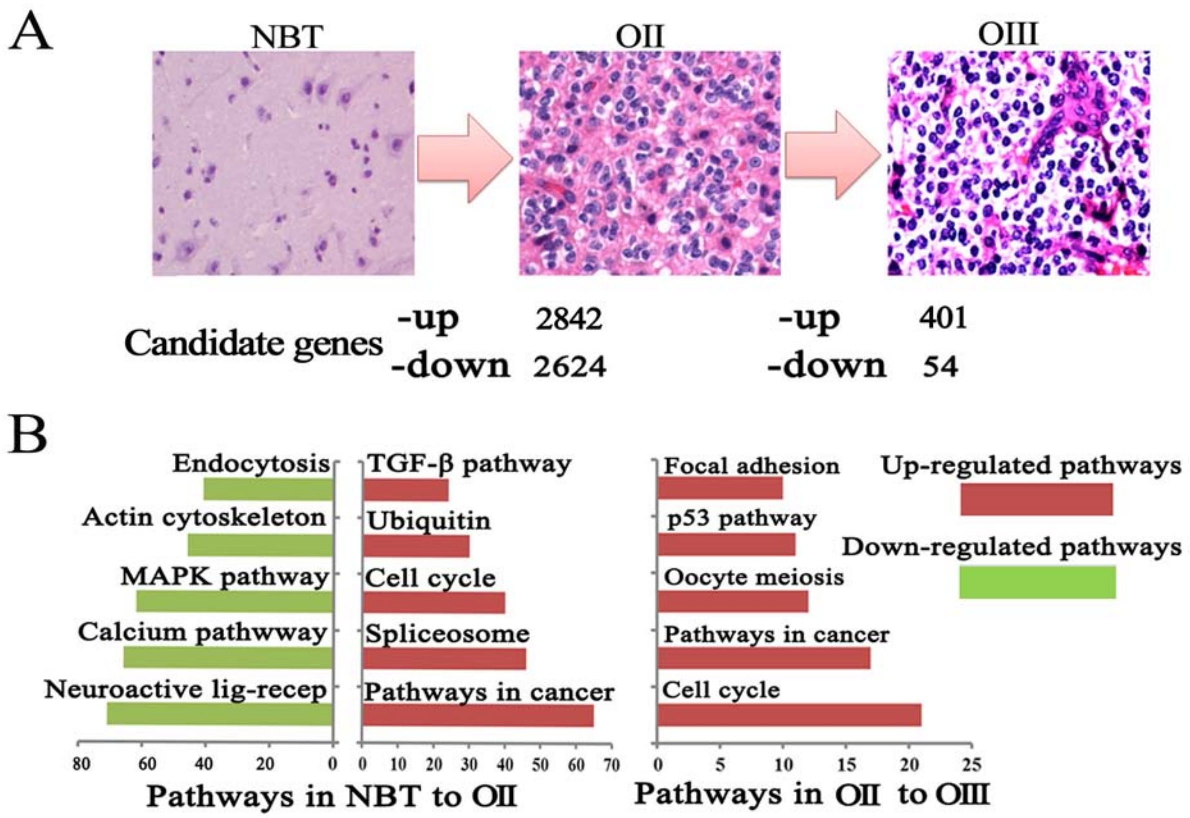

Supplementary Figure S2: Number of differentially expressed genes (upregulated and downregulated) with increasing tumor grades. A. These genes were identified by analysis of the overlapping data from the three datasets ($p < 0.05$). **B.** Gene-set enrichment analysis was performed using a functional annotation tool (DAVID).

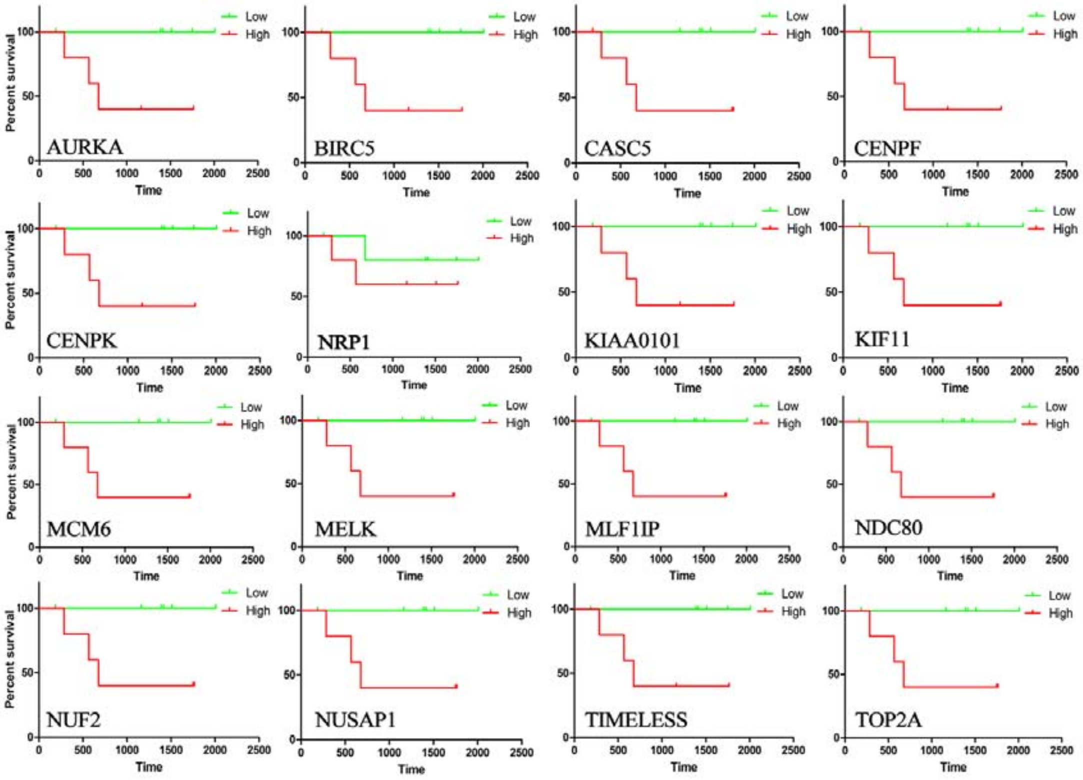

Supplementary Figure S3: Kaplan–Meier survival analysis identifying the correlation between the sixteen candidate genes and the survival of patients with OIII based on the CGGA dataset (11 OIIIs).

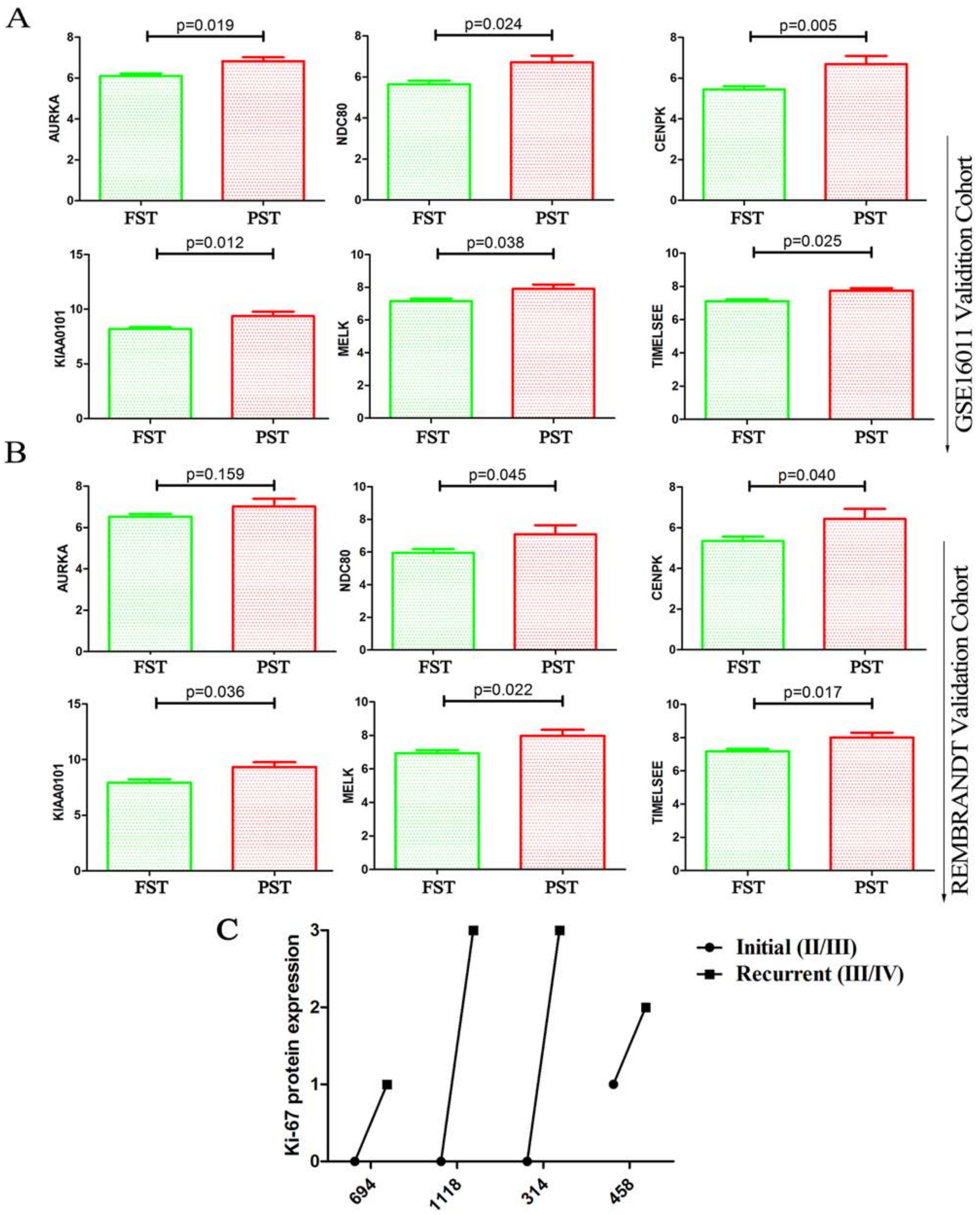

Supplementary Figure S4: Candidate genes were associated with TCGA subtypes. A. and **B.** The relationship between the candidate genes and the TCGA subtypes was validated on the GSE16011 and REMBRANDT datasets. **C.** The recurrent tumors contained the ki-67 protein is higher than those observed in the initial tumors ($p = 0.02$)

Supplementary Table S1: The numbers of samples with microarry and RNA-seq

Datasets	Microarray (number)			Total	RNA-seq (number)		Total
	NBT	OII	OIII		OII	OIII	
CGGA	5	17	11	33	27	11	38
GSE16011	8	8	44	60	–	–	
REMBRANDT	21	30	23	74	–	–	
TCGA	–	–	–	–	64	41	105
Total	34	55	78	167	–	–	–

NBT: Normal Brain Tissue; OII/III: Oligodendroglioma grade II/III

Supplementary Table S2: The list of differentially expressed genes in three datasets.

See Supplementary File_S1

Supplementary Table S3: The primers of candidate genes

Genes	Forward Primer (5′ → 3′)	Reverse Primer (5′ → 3′)
AURKA	CAGGCAACCAGTGTACCTCATCC	GGCGACCAATTTCAAAGTCTTCC
NDC80	AAATCAAGGACCCGAGACCACT	GGCACAGGAAGCCATAAAGAAA
KIAA0101	CTAATTCGACATCAGTTTCATCG	TCAGAATCTTTAGGGGACAACC
TIMELESS	TGAGGAAGACAGCGAAGAGGAA	GAGCCATAGGAGCGGGATAGAA
MELK	TGTTGAGTGGCAAAGCAAGAATC	AGCAGAAGATAGGTAGCCGTGAG
CENPK	AAACACTCACCGATTCAAATGC	GAGAACGTCTTCAGTCAAGGGA
ACTIN	TGACCCAGATCATGTTTGAGA	TACGGCCAGAGGCGTACAG
